# Supplementary material for: Transscleral photodynamic therapy with a chlorin e6: An experimental study of exposure parameters and therapeutic window
Source: PLoS One. 2026 Jan 28;21(1):e0341058. doi: 10.1371/journal.pone.0341058 (PMC12851484; doi:10.1371/journal.pone.0341058)
Supplement: Attachment — Submitted filename: Comment Plos 10052025.docx [file pone.0341058.s002.docx]

**Comments and Queries from Reviewer**

The topic of article presented is appreciative and study reports certain worthwhile information that can be considered useful for clinical translation in future. Data appears detailed and thorough. The author appears to have an appreciative effort in discussion section to provide thorough arguments regarding the study as well as its drawback and future prospects in discussion. Thus, it is a useful study to be reported. Although, there are a few suggestions and queries for the author, which are as follows:

1. <https://journals.sbmu.ac.ir/jlms/article/view/47901/35787> (Transscleral Photodynamic Therapy with a Chlorin e6 Photosensitizer in a Rabbit Experimental Model of an Intraocular Mass Lesion: TS-PDT in experimental ocular mass lesion model).
   a. The reviewer suggests the author to explain if this study is different from the given manuscript. If possible please share the manuscript PDF, as it is not accessible.
2. In the Line 179-181 (The therapeutic window and key parameters (laser power, power density, energy density, thermal dynamics) were first-ever systematically defined by correlating clinical, histological, and thermal responses in a controlled model)
   a. Is the author sure about the claims mentioned above? Please also shed some light and explain about the clinical correlation other wise the author may modify the statement.
3. Twenty-one rabbits (3.0–3.5 kg) underwent ophthalmic screening. Procedures followed the 69 Declaration of Helsinki and Directive 2010/63/EU, with approval from the local Ethics Committee 70 of the Federal State Budgetary Scientific Institution “IEM” (Protocol No. 4/24, dated October 24, 71 2024).
   a. This paragraph and like this other major content of the article has come under plagiarism indicating use of some AI software. The reviewer advise to the author to kindly re-check whole manuscript for the AI plagiarism and paraphrase the text.
4. The manuscript needs to review by author for reference citation correction (for example reference 5 or 6 doesn’t suggest correct citation for line 205, and First sentence specific reference needed instead of adding reference after paras.) likewise there are more. The references are at certain places not accurate or wrongly added.
5. Material method 2nd para lacks sentences wise specific references. Rather bunch of references added altogether after couple of sentences. This confuses readers to choose correct reference for specific context.
6. The reviewer suggests the author to kindly cite references next to each sentence where it is referred to, instead of citing a bunch of references after 2-3 sentences combined. It is only confusing the readers and not giving accurate information.
7. Introduction section lacks streamlined discussions rather puts useful information in random order in a way that the discussion topics abruptly shifts from one to another without any order. The author wants to share useful information but it needs to streamlined and described in orderly manner while connecting the dots as a storyline discussion.
8. Which species of rabbits selected for animal study?
9. This sentence is unclear. This sentence has missing to write “treatment Zones”.
   a. Due to the rabbits’ globe size, two zones were possible with the 10 mm probe and three zones with the 5 mm probe.
10. The following sentences appears to be unclear. How 10 Rabbits have 20 right eyes or other 10 rabbits have 20 left eyes.
    a. Rabbits were divided into two groups:

Group I (5 mm probe, 10 rabbits, 20 right eyes): 0.1 W, 0.17 W, 0.3 W; exposure 600 s. Group II (10 mm probe, 10 rabbits, 20 left eyes): : 0.3 W, 0.6 W; exposure 600 s.

11. Is the author sure to use the term “Clinical” in the abstract under the purpose section for “animal-based study”? if yes, please justify.
12. Among the three zones of used in the study using 5 mm probe, two (0.1 W, and 0.17 W) are discussed although, the discussion on zone 3 (0.3 W) appears to be lacking.
13. Histological changes on 10 mm probe of 0.3 W in fig 2C is missing.
14. Please explain in line 148 author mentioned the p value as p< 0.001, however, in the table 2 group 2 it is shown as p= 0.1, which one is correct?
15. The captions for fig 3A and Fig 3B are same!?
16. Table 3 column 5 heading show T10 – T0, but T5 – T0 is not added in the table, please explain the reason. What is the clinical significance of Scleral Temperature Changes? Please answer both queries separately.
