# Supplementary material for: Transscleral photodynamic therapy with a chlorin e6: An experimental study of exposure parameters and therapeutic window
Source: PLoS One. 2026 Jan 28;21(1):e0341058. doi: 10.1371/journal.pone.0341058 (PMC12851484; doi:10.1371/journal.pone.0341058)
Supplement: Attachment — Submitted filename: Plos OneResponse to Reviewer 2 (PONE-D-25-24966).docx [file pone.0341058.s003.docx]

**Dear Reviewer,**

The author team would like to express our sincere gratitude for your positive assessment and constructive comments on our manuscript. We have carefully analyzed your feedback and made corresponding corrections. Below we provide a point-by-point response to your questions.

| **Reviewer's Comment** | **Authors' Response and Actions Taken** |
| --- | --- |
| 1. **The question is: how can 10 rabbits have 20 right eyes or 20 left eyes? I believe this is not correct. Please review/clarify.** | We apologize for the confusion in the initial description of the study design. The study design was as follows: 21 rabbits participated in the study. Twenty of them received bilateral treatment (both eyes):  - The right eye of each of these 20 rabbits was assigned to Group I (5 mm probe).  - The left eye of each of these 20 rabbits was assigned to Group II (10 mm probe).  - One rabbit (one eye) constituted the control group (no exposure).   Thus, the total number of eyes was: 20 (right, Group I) + 20 (left, Group II) + 1 (control) = 41 eyes. This clarification has been added to the "Materials and Methods" section of the manuscript:   **"Rabbits were divided into two groups. Among the 21 rabbits, 20 received bilateral treatment: their right eyes were assigned to Group I (5 mm probe, 20 eyes) and their left eyes to Group II (10 mm probe, 20 eyes). The remaining one rabbit (one eye) served as a control. In Group I, three different power settings were applied to distinct zones: 0.1 W, 0.17 W, and 0.3 W, with an exposure time of 600 s for each. In Group II, two power settings were used: 0.3 W and 0.6 W, also with 600 s exposure for each zone."** **[(Line 102-109)]** |
| 2. **Tables legends must describe the statistical tests used.** | We thank the reviewer for this important remark. We have revised all table legends to include a description of the statistical tests used, ensuring they correspond to the methods described in the "Statistical Analysis" section of the manuscript.   The specific changes made are as follows:  - **Table 1:** The legend now includes: "Statistical analysis was performed using the χ² test." **[(Line 143)]** - **Table 2:** The legend now includes: "Statistical analysis was performed using the χ² test." **[(Line 159)]** - **Table 3:** The legend now includes: "Statistical analysis was performed using ANOVA and Student's t-test." **[(Line 187)]**  These additions ensure clarity and guarantee that the statistical methodology is transparently reflected for each table, as recommended. |
| 3. **The main limitations of the study are the use of healthy rabbit's eye to simulate the application of therapy aimed at tumor treatment. The model does not simulate the tumor microenvironment and a deeper discussion on the possible clinical application of the methodology presented in a healthy animal model should be provided. A deeper discussion on the main challenges of the study for a clinical application should be provided.** | We thank the reviewer for this comment regarding the limitations of our healthy eye model and the need for a deeper discussion of clinical translation. We have significantly expanded the corresponding section in the "Discussion" to more thoroughly address these important points.   The revised text now includes:  1. A more detailed acknowledgment of the specific limitations of using healthy eyes compared to tumor-bearing eyes.  2. A clearer explanation of why this fundamental study was necessary despite these limitations.  3. A more comprehensive discussion of the specific challenges for clinical translation.  4. Specific steps and technical developments needed to bridge the gap between experimental data and clinical application.   We believe these additions significantly strengthen the "Discussion" section and provide readers with a more realistic perspective on both the value and the limitations of our work. **[(Page 11)]** |
